# Novel Functions of Integrins as Receptors of CD154: Their Role in Inflammation and Apoptosis

**DOI:** 10.3390/cells11111747

**Published:** 2022-05-25

**Authors:** Ghada S. Hassan, Suzanne Salti, Walid Mourad

**Affiliations:** Laboratoire d’Immunologie Cellulaire et Moléculaire, Centre de Recherche du Centre Hospitalier de l’Université de Montréal (CR-CHUM), 900 rue Saint-Denis, Tour Viger, Montréal, QC H2X 0A9, Canada; ghada.s.hassan@gmail.com (G.S.H.); suzanne.salti@umontreal.ca (S.S.)

**Keywords:** integrins, CD154, active/inactive conformation, receptor–ligand binding, inflammation, apoptosis, malignancy

## Abstract

CD154, an inflammatory mediator also known as CD40 ligand, has been identified as a novel binding partner for some members of the integrin family. The αIIbβ3, specifically expressed on platelets, was the first integrin to be described as a receptor for CD154 after CD40. Its interaction with soluble CD154 (sCD154) highly contributes to thrombus formation and stability. Identifying αIIbβ3 opened the door for investigating other integrins as partners of CD154. The αMβ2 expressed on myeloid cells was shown capable of binding CD154 and contributing as such to cell activation, adhesion, and release of proinflammatory mediators. In parallel, α5β1 communicates with sCD154, inducing pro-inflammatory responses. Additional pathogenic effects involving apoptosis-preventing functions were exhibited by the CD154–α5β1 dyad in T cells, conferring a role for such interaction in the survival of malignant cells, as well as the persistence of autoreactive T cells. More recently, CD154 receptors integrated two new integrin members, αvβ3 and α4β1, with little known as to their biological significance in this context. This article provides an overview of the novel role of integrins as receptors of CD154 and as critical players in pro-inflammatory and apoptotic responses.

## 1. Introduction

Integrins are transmembrane adhesion receptors that have crucial functions in cell adhesion, survival, proliferation, and many other highly significant processes in normal and disease states. They are a family of heterodimeric receptors mediating cell interactions, anchorage, and migration. In mammals, 18 α and 8 β subunits combine to form 24 integrins [1]. Alternative splicing [2] and post-translational modifications [3] ensure a high diversity in integrin structure. Such diversity in subunit composition contributes to their expanded ligand recognition, binding to the extracellular matrix (ECM), and coupling to downstream signaling pathways [4,5,6,7,8,9]. They bind to ECM proteins, transmembrane adhesion molecules, glycoproteins in circulation, and other ligands. Interestingly, CD154, which is a co-stimulatory molecule belonging to the tumor necrosis factor (TNF) family, has been identified as a new integrin ligand. CD154 binds to several integrins—namely, αIIbβ3 [10], αMβ2 [11], α5β1 [12], αvβ3 [13], and α4β1 [14].

## 2. CD154

**Figure 1:**
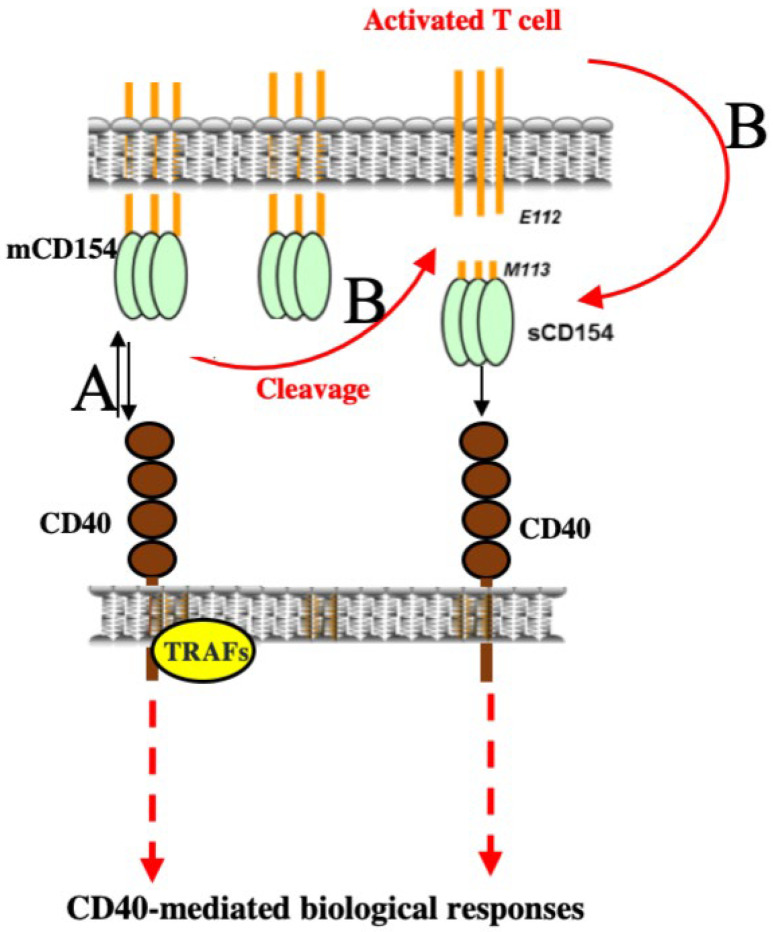
The bidirectional interaction of CD154 with CD40 induces CD154 cleavage and CD40-mediated biological responses. CD154 expressed on the surface of T cells (membrane-bound, mCD154) is capable of binding to CD40 and inducing a bidirectional intracellular signaling cascade and subsequent biological responses in both CD40-positive and CD154-positive cells (**A**). In order to put a halt to the CD154–CD40 biological response, CD154 expressed on the surface of T cells is cleaved into soluble CD154 (sCD154) upon its binding to CD40. Soluble CD154 may be also released from the intracellular milieu (**B**). Both mCD154 and sCD154 are capable of binding and activating CD40 inducing biological responses in CD40-positives cells. Engaging CD40 induces recruitment of TRAFs, i.e., TRAF1, TRAF2, TRAF3, TRAF5, and TRAF6, and subsequent triggering of intracellular signaling events. Abbreviation: TRAF, TNFR-associated factor.

CD154 is a 33–39 kDa type II transmembrane protein composed of an extracellular region of 215 amino acids (AAs), a 24-AA transmembrane domain, and a 22-AA intracellular tail [15]. CD154 is mainly expressed on the surface of activated T cells and platelets but has also been described in mast cells, basophils, and eosinophils [15,16]. Monomeric CD154 molecules non-covalently link to each other, forming a homotrimer, which is of absolute importance for CD154 biological function [17,18,19]. Intracellular and membrane-bound CD154 (mCD154) molecules are prone to cleavage, releasing a soluble form involving residues 113-261, which is biologically active (trimeric) [20]. Studies by various groups, including ours, have demonstrated that soluble CD154 (sCD154) is released from activated platelets and T cells by proteolytic cleavage at its methionine residue at position 113 (M113), mediated by various members of the metalloproteinases family and initiated by the interaction of the ligand with CD40 in cleavage from T-cell surface (Figure 1) and/or with αIIbβ3 in platelets cleavage [20,21,22,23,24,25]. Although the physiological role of sCD154 in vivo remains unclear, increased levels were reported in many inflammatory disorders [26,27,28]. In addition, it has been proposed that cleavage of CD154 from the cell surface serves to attenuate the CD40-mediated immune response since mCD154 was shown to exhibit stronger effects in CD40-positive cells than its soluble counterpart [29,30,31]. Indeed, while mCD154 expressed on platelets was capable of promoting activation of endothelial cells (ECs) by inducing and/or upregulating their surface expression of adhesion molecules such as E-selectin, vascular cell adhesion molecule-1 (VCAM-1), and intercellular adhesion molecule-1 (ICAM-1), and their release of IL-8 and monocyte chemoattractant protein-1 (MCP-1), its soluble counterpart failed to stimulate such effect. Furthermore, mC1D54 but not sCD154 was capable of inducing apoptosis of CD40-positive malignant cells [29,30]. Later studies, including ours, provided more proof as to the higher potency of mCD154 in inducing signaling and biological responses in CD40-positive cells, thus outlining the importance of the CD154 cleavage process in attenuating CD40-mediated effects [24,31]. In addition to confirming this hypothesis [24], our recent observations strongly suggest that cleavage of CD154 into sCD154 is also necessary for binding to certain integrin receptors [32]. As a matter of fact, most studies that demonstrated an interaction between CD154 and integrins used the soluble form of the latter [10,11,12,13,14]. In the case of α5β1, our data showed that membrane-bound CD154 is capable of interacting with the integrin only when both molecules are expressed on the same cell surface, i.e., a cis-type of interaction (discussed in detail below) [32]. Preliminary data from our lab have shown that cells expressing the αIIbβ3 or αMβ2 (also known as Mac-1) integrins on their surface were not capable of interacting with other cells expressing CD154 (CD154-transfected Jurkat E6.1 T cells). However, a previous study by Wolf et al. in 2011 suggested that Mac-1 could interact with CD154 expressed on the cell surface [33]. Thus, additional studies are now in place by our group to further characterize this issue. It is essential to point out here that even though we, and probably others, failed to demonstrate binding between mCD154 and integrins expressed on the cell surface, this does not exclude the existence of such binding, probably at a low binding affinity, hence the need for deeper research into this type of interaction.

## 3. CD40: The Classical CD154 Receptor

CD40 is a 45–50 kDa phosphorylated type I integral membrane glycoprotein initially regarded as the only receptor for CD154 [15]. It is expressed on a variety of immune and non-immune cells including B lymphocytes, monocytes, macrophages, dendritic cells (DCs), fibroblasts, endothelial and epithelial cells, etc. [15,16,34,35]. The primary function of CD154 was believed to be only linked to humoral immunity via its interaction with CD40 [36]. Today, it is well-established that CD154 has a larger role than originally described, as it is involved in a variety of inflammatory immune responses [15,35].

CD40 is a transmembrane protein lacking direct intracellular enzymatic activity, hence its dependence on adaptor molecules for the proper relay of signals inside the cell, a function ensured by TNFR-associated factors, TRAFs. TRAFs 1, 2, 3, 5, and 6 have been shown to work with CD40 in its cell activation mission. Upon ligation of CD40 and its subsequent recruitment of TRAFs, several signaling cascades are triggered, activating nuclear factor κ B (NFκB), mitogen-activated protein kinases (MAPKs) p38, and extracellular signal-regulated kinase 1/2 (ERK1/2), phosphatidylinositol- 3 kinase (PI-3K), Akt, c-Jun N-terminal kinase (JNK), STAT3, etc. [34,37,38,39,40]. Bidirectional signaling has been established upon ligation of mCD154 to CD40, which elicits responses in both CD40-positive and CD154-positive cells—namely, T cells (Figure 1) [24,41,42,43,44,45,46].

By engaging CD40 on the surface of B cells, CD154 was shown to co-stimulate and enhance activation and maturation of these cells, inducing, together with IL-4 produced by T cells, the formation of germinal centers, antibody production, and Ig switching. The importance of CD40–CD154 interaction in T-cell-dependent B-cell responses in vivo emerged when point mutations or deletions in the gene coding for CD154 were shown to cause X-linked hyper IgM syndrome (HIGM) [47]. Patients suffering from this syndrome have a normal number of circulating B cells but low levels of serum IgG, IgA, and IgE and elevated IgM. As a result, they develop an increased susceptibility to various infections (reviewed in [47]). In addition, the CD154–CD40 interaction is involved in cell-mediated immunity by activating monocytes, macrophages, ECs, DCs, fibroblasts, and other types of cells, upregulating their co-stimulatory and adhesion molecule expression and their cytokine release [48,49]. Engaging CD40 on macrophages promoted their pro-inflammatory phenotype by enhancing their production of inflammatory mediators such as IL-1β, IL-6, IL-12, TNF-α, MCP-1 as well as matrix metalloproteinases (MMP)-2 and MMP-9 [50,51]. Similarly, CD40-mediated effects in DCs included upregulating their release of IL-1, IL-6, and IL-12 cytokines [52,53]. Additional target cells of CD154, specifically in the vasculature, include ECs. The stimulation of ECs with CD154 enhances their production of a plethora of cytokines/chemokines, including IL-6, IL-8, IL-15, MCP-1, MCP-3, macrophage inflammatory protein-3 (MIP-3) and regulated upon activation, normal T-cell expressed, and secreted (RANTES), highly contributing to the role of the endothelium in atherogenesis [51,54,55]. In addition to this inflammatory function in the context of cytokine enhancement, CD154–CD40 dyad upregulates the expression of activation markers and adhesion molecules on the surface of macrophages and DCs—namely, CD80/CD86, ICAM-1, and LFA-3, on the surface of ECs (E-selectin, ICAM-1, and VCAM-1), as well as other cell types [15,56,57,58,59,60]. All these findings outline an important signature for the CD154–CD40 dyad in a wide range of inflammatory responses [15,27,35], some of which lead to the pathogenic process of chronic inflammatory autoimmune diseases, including rheumatoid arthritis, systemic lupus erythematosus, multiple sclerosis [61], autoimmune thyroiditis [62], Sjögren’s syndrome [63], type I diabetes [64,65], polymyositis, dermatomyositis [66], leprosy [67], inflammatory bowel diseases such as Crohn’s disease and ulcerative colitis [68], as well as vascular diseases such as atherosclerosis and restenosis [48,49,65,69].

In addition to the above-described functions, there is growing evidence to support the role of the CD154–CD40 dyad in cancer immunotherapy [70]. The antitumor effect of this axis was first demonstrated in studies showing that co-culturing CD4-positive T cells expressing CD154 with DCs leads to maturation of these latter and an enhancement of their antigen (Ag)-presentation capacity [70,71,72]. Subsequently, CD154-stimulated DCs can activate CD8-positive cells and their cytotoxic functions against tumor cells. A more direct role of CD154 in cancer regulation is represented by its capacity to halt growth or induce apoptosis of malignant cells via its interaction with CD40 on their surface [29,73]. Indeed, sCD154 was shown to inhibit the proliferation of CD40-positive urothelial cells of normal or malignant origin, while the membrane-bound ligand induced apoptosis in these cells [74]. Ligating CD40 on B cells from higher grade malignancies such as large cell lymphoma of B-cell origin or Epstein-virus-induced B-cell lymphoma arrested their growth and even reduced tumor development in animal tumor models [75]. The apoptotic function of CD40 via its interaction with membrane-bound ligands was shown to involve the upregulation of TRAF3/TRAF6 and activation of JNK and activator protein-1 (AP-1), ERK1/2, caspases 3, 8, and 9 [76,77]. It is worth noting here some evidence pointing to the rather potential causative role of CD154 in tumor development. Indeed, CD154 was shown to induce the proliferation of some malignant cells and boost their immunogenicity, motility, and metastatic capacity [78,79,80]. Mechanisms involved in the tumor-promoting effect of the CD154–CD40 dyad include activation of NF-κB and enhancing the production of angiogenic/growth factors such as platelet-activating factor (PAF) [78,79,80,81].

All these data and observations outline the critical role played by CD154 in immune, inflammatory, and immunotherapeutic responses by interacting with its classical receptor, CD40 (Figure 1). However, the story becomes more complicated with the discovery of additional receptors for CD154, all members of the integrin family.

## 4. Novel Receptors of CD154

**Figure 2:**
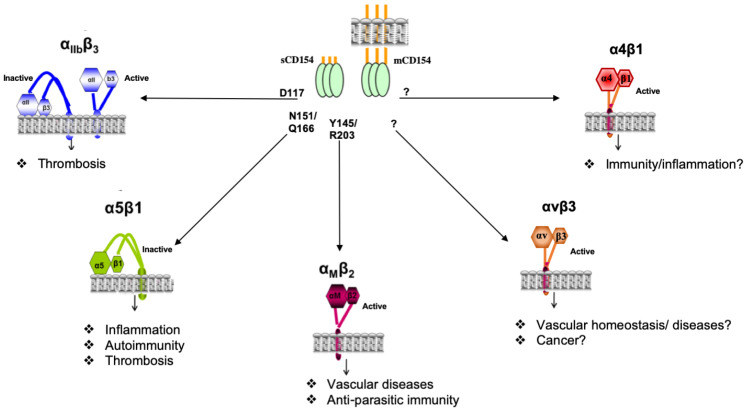
Model representing the interaction of CD154 with integrins. CD154 is capable of binding to its receptors belonging to the integrin family, the αIIbβ3, α5β1, αMβ2, αvβ3, and α4β1 integrins, in their active and/or inactive forms depending on the integrin. Distinct residues of CD154 are implicated in its interaction with integrins. The CD154–integrin binding participates in various biological responses and as such is involved in the pathology of numerous disease conditions.

It is now well-established that CD154 binds to receptors other than CD40, belonging to the integrin family—namely, the αIIbß3 [10], αMβ2 [11], α5β1 [12], αvβ3 [13], and α4β1 [14] integrins, and to be implicated as such in the pathogenesis of multiple diseases and disorders (Figure 2).

### 4.1. Structural Interaction of CD154 with Its Receptors

The interaction of CD154 with its receptors was analyzed by site-directed mutagenesis [82] and co-crystal analysis [83]. Residues of CD154 involved in its binding to CD40 were defined as K143, Y145, Y146, R203, and Q220 [82]. According to André et al., CD154–αIIbβ3 interactions are mediated by the RGD residues of murine CD154 and the KGD residues of human CD154 (at positions 115-117) [10,84]. Upon substitution with alanine (A) of the CD154 D117 residue, the CD154–αIIbβ3 interaction was abrogated, suggesting that D117 is the major binding residue implicated herein. Although our observations demonstrated the ability of CD154 to bind the α5β1 integrin, they do not support the involvement of the KGD domain in this interaction [12]. To identify the CD154 residues involved in α5β1 and αMβ2 binding, a panel of CD154 mutants has been generated [85] and used for functional and binding analyses [12,86]. Our data showed that CD154 ligation to α5β1 is totally independent of its binding to αIIbβ3 and CD40 molecules [86,87] and that residues N151 and Q166 of CD154 are required for α5β1 binding, whereas CD154 residues required for the interaction with the αMβ2 integrin, Y145, and R203 are shared with those involved in CD40 binding [86]. CD154 residues implicated in the binding with very recently identified receptors—αvβ3 and α4β1 integrins—have not yet been identified, even though an integrin-binding site in the trimeric interface of CD154 has been suggested [13]. Although all belong to the integrin superfamily, CD154 was capable of binding to its relatively new receptors in different states of their activation. While it was shown to interact with inactive α5β1 and with active αMβ2, αvβ3, and α4β1, CD154 bound to both inactive and active forms of αIIbβ3 [10,11,12,13,14] (Figure 2).

### 4.2. The αIIbβ3 Integrin as a Receptor of CD154

Belonging to the group of RGD-recognizing integrins and binding to RGD-containing peptides including fibrinogen, αIIbβ3 integrin, mainly expressed on platelets and their precursors, megakaryocytes, is a critical element in the process of platelet aggregation. In 2002, αIIbβ3 was identified as a receptor for CD154 [10]. The authors demonstrated the direct binding of the ligand to αIIbβ3 via the D117 residue in the KGD region of human CD154 (RGD in murine), inducing platelet spreading and allowing platelet–platelet interaction [10]. Using CD154 knockout (KO) mice and their wild-type (WT) littermates, CD154 was shown to be involved in thrombus stabilization, as evidenced by the increased thrombi rupture in injured arterioles of CD154 KO mice, owing to their low platelet content, compared with WT ones. Furthermore, treatment of CD154 KO platelets with recombinant sCD154 enhanced their thrombin-induced aggregation under high sheer stress conditions [10]. The same group subsequently investigated signaling events underlying the CD154–αIIbβ3 interaction and demonstrated the capacity of sCD154 to induce tyrosine phosphorylation of the integrin β chain and the abrogation of such signal in the presence of antibodies directed against β3 tyrosine phosphorylation or in mice with mutated β3 tyrosine residues. As such, by activating the integrin, CD154 promoted outside-in signaling in platelets, the formation of platelet microparticles, and enhancement of fibrinogen binding [88]. Soluble CD154 was later shown to enhance the production of reactive oxygen and nitrogen species in platelets via binding to αIIbβ3 in a mechanism involving activation of p38 MAPK as well as Akt [89]. Another study rather aiming at characterizing the CD154–CD40 dyad in platelet signaling outlined a role of CD154 in activating PI-3K and Akt independently of CD40 and probably involving the other CD154 receptor on platelets, the αIIbβ3 integrin [90]. The relationship between CD154 and the αIIbβ3 integrin was further underlined by the finding that ligand-induced activation of αIIbβ3 upregulates expression of CD154 on the surface of platelets, promoting in this matter their crosstalk with CD40-expressing endothelial cells, a critical phenomenon in vascular injuries [91]. Unlike other ligands, CD154 binds to active and inactive forms of αIIbβ3 [10,88], highlighting the significance of the CD154–αIIbβ3 interaction under various conditions, including static ones.

### 4.3. The αMβ2 Integrin as a Receptor for CD154

The αMβ2 integrin, a member of the leukocyte-specific or LDV-binding group of integrins [92], is mainly expressed on cells of the myeloid lineage, including monocytes, macrophages, and granulocytes, and of lymphoid descent such as natural killer (NK cells) [93]. It binds to a variety of ligands, such as C3bi, intercellular adhesion molecule-1 (ICAM-1), heparin, fibrinogen, vitronectin, fibronectin, etc. [94,95,96,97,98,99]. This integrin, also known as Mac-1, is implicated in the adhesion of myeloid cells to the ECM upon interaction with one of its ECM ligands. In addition, Mac-1 is mainly involved in the adhesion and rolling of myeloid cells and, more specifically, monocytes onto ECs via its binding to adhesion molecules such as ICAM-1 [100], receptor for advanced glycation end-products (RAGE) [101] and endothelial protein C receptor (EPCR) [102] usually expressed on the latter cell type. In 2007, Zirlik et al. identified CD154 as a new ligand of Mac-1 [11]. Using monocytes and Mac-1-transfected Chinese hamster ovary (CHO) cells, it was shown that CD154 induces activation of these cells, their migration, their adhesion even under sheer conditions, and their myeloperoxidase (MPO) release by binding to their Mac-1, especially the active form of the molecule. The authors also reported the role of the CD154–Mac-1 dyad in recruiting monocytes into the peritoneal injury site in vivo and further outlined the role of such interaction in atherosclerosis development using mouse models of the disease [11]. It is important to mention here that the role of Mac-1 in vascular abnormalities has been demonstrated way before its identification as a receptor for CD154 and more as a receptor for different ECM-associated proteins, initially in an angioplasty setting and later in atherosclerosis development [103,104,105]. Thus, the implication of Mac-1 in vascular diseases involves several mechanisms and the interaction with different types of ligands.

Back to the CD154–Mac-1 dyad, more recent studies have recognized the I domain of the αM chain, involved in the binding of the Mac-1 integrin to many ligands, as also mediating its interaction with CD154 [33]. A binding epitope (E^162^-L^170^) has been identified in this matter using a set of peptides designed to match various areas within the αM I domain, with one of the peptides capable of binding to labeled CD154 and specifically inhibiting the interaction of CD154 with Mac-1-expressing cells. Using this inhibitory peptide, the role of the CD154– Mac-1 dyad was further confirmed in leukocyte adhesion and rolling onto the endothelial layer, peritoneal inflammation, and even atherosclerotic lesion development and stability [33]. The same group later demonstrated the efficiency of a monoclonal antibody (mAb) directed against the E^162^-L^170^ epitope of Mac-1 in specifically abolishing leukocyte recruitment without affecting other integrin-related responses [106]. This inhibitory peptide has more recently been used to outline yet another signature of the CD154–αMβ2 interaction as a mediator of allograft rejection [107]. Treatment of engrafted mice with the inhibitory peptide increased their graft survival, attenuated their CD8-positive- and innate-mediated alloimmunity, and even potentiated the allograft-protecting effect of an antagonistic anti-CD40 Ab. These findings suggest a crosstalk between both CD154 receptors—Mac-1 and CD40—in alloimmunity [107].

In spite of the growing interest in the CD154–Mac-1 interaction and its role in vascular diseases and immune pathologies, little is known about the underlying signaling events. Since CD154 was shown to bind to the active form of Mac-1 [11], the intracellular proteins that are usually recruited by β2 integrins, ensuring their activation and association with the cytoskeleton, including Talin-1 and Kindlin-3 [108,109], should be already in their integrin-binding position when CD154 interacts with Mac-1. Similar to other ligands, engaging Mac-1 with CD154 might involve the downstream activation of Src-family tyrosine kinases, hematopoietic cell kinase (HCK), and spleen tyrosine kinase (SYK) [109,110]. Such areas of research await further exploration.

In addition to the ligand–receptor interaction, a different kind of relationship exists between CD154 and Mac-1. Indeed, by interacting with its CD40 receptor on neutrophils, CD154 was shown to enhance the expression as well as a PKCζ-dependent activation of Mac-1 in these cells, mediating their adhesion to platelets and transmigration through a platelet layer in vitro or in a denuded vessel in restenosis mouse models [111,112].

Furthermore, recent data have shown a role for the CD154–αMβ2 dyad in IL-12 production and Th1 immune responses against *Leishmania major* infections [113]. Researchers have shown that CD154 KO mice and not CD40 KO ones exhibited reactivation of their infection due to a reduced IL-12 and IFN-γ production by their DCs and monocytes in a Mac-1-dependent manner. The protection of CD40 KO mice against reinfection was abolished upon their treatment with anti-Mac-1 antibodies [113].

Altogether, these data identify yet another member of the integrin family—Mac-1, also known as αMβ2—as a receptor for CD154 and outline a new pathological signature of this latter in vascular diseases.

### 4.4. The α5β1 Integrin as a Receptor for CD154

The α5β1 integrin belongs to the subfamily of RGD-recognizing integrins [92]. Numerous types of cells express the α5β1 integrin on their surface, including T and B cells, platelets, monocytes, endothelial and epithelial cells, etc. [114,115]. Upon binding to its natural ligand, fibronectin, the α5β1 integrin promotes several cellular functions involving adhesion, proliferation, survival, and motility [114,115]. Based on studies showing that the absence of CD154 but not CD40 protected mice against bronchial hyperresponsiveness [116], that treating monocytes with sCD154 upregulated their tissue factor expression independently of CD40 [117], and that a CD154–αIIbβ3 dyad is involved in platelet activation and stabilization of thrombus [10], our group further investigated into the interaction of CD154 to yet other receptors. We reported that sCD154 is capable of binding to a CD40-negative/α5β1-positive (and obviously αIIbβ3-negative) monocytic cell line [12]. The binding of sCD154 to these cells was inhibited by soluble α5β1 and anti-α5β1 mAb, whereas the binding of sCD154 to CD40-positive B cells was only inhibited by an anti-CD40 mAb [12]. Engaging α5β1 with sCD154 activated signaling molecules including the ERK1/2 MAPK and upregulated IL-8 gene expression. Interestingly, sCD154 induced translocation of α5β1 to lipid rafts, ensuring their communication to the cell cytoskeleton. Soluble CD154 interaction with monocytes was shown to involve the inactive conformation of α5β1 integrin since activation of α5β1 by Mn^2+^ or DTT that leads to an increase in the binding of α5β1 to its natural ligand fibronectin [118], resulted in a decreased ligation of α5β1 integrin by sCD154 [12].

While CD154-induced response in α5β1-positive monocytes could be prevented by soluble α5β1, the resulting activation of intracellular signaling in CD40-positive B cells was not affected, suggesting that sCD154 may bind concomitantly to both CD40 and α5β1. Indeed, and as mentioned above, our group subsequently demonstrated that engaging simultaneously CD40 and α5β1 with specific mAbs activated p38 and ERK1/2 MAPK and concurrently enhanced the production of inflammatory factors such as MMP-2 and -9, suggesting a high level of communication between both receptors [87].

In a recent study further investigating the interrelation between α5β1 and CD40 using a docking model, it was reported that α5β1 might be interacting with monomeric CD154 at the interface of its trimeric structure [13]. In accordance with the earlier study that described α5β1 as a receptor for CD154 [12], the CD154–α5β1 binding outlined herein was also shown to be independent of the KGD motifs of CD154; however, it involved an interaction with the active form of the integrin. Mutating this predicted binding region in CD154 inhibited its interaction with α5β1 and the subsequent antiapoptotic function. In addition, this CD154 mutant, while still capable of binding to CD40, exhibited an abrogated CD40-mediated signaling and responses in B cells. Thus, researchers have suggested the existence of a CD40–CD154–α5β1 ternary complex [13]. In addition, these findings further leveled up the role of the CD154–α5β1 dyad and suggested its implication in the pathogenesis of hyper IgM syndrome, as some of the mutations characterizing this disease condition fall within the CD154 trimeric interface region involved in the interaction with the integrins [13,14].

#### 4.4.1. Inflammatory Function of the CD154–α5β1 Dyad

Initial studies identifying α5β1 as a new receptor for CD154 demonstrated the role of this dyad in pro-inflammatory responses [12]. The CD154–α5β1 interaction was shown to induce ERK1/2 phosphorylation in monocytes, promoting their pro-inflammatory functions and release of IL-8 [12]. The crosstalk between T cells and asthmatic fibroblasts via their CD154 and α5β1, respectively, promoted IL-6 release and could be also responsible for upregulating the binding of these fibroblasts to fibronectin [119]. A further role of the CD154–α5β1 dyad in inflammation was demonstrated as part of a synergistic response with a CD154–CD40 interaction, enhancing intracellular MAPK activation and the release of inflammatory mediators, including MMP-2 and MMP-9, as mentioned above [87].

Since platelet activation has been recently categorized as part of the inflammatory process [120,121], the following observations add to the inflammatory functions of the CD154–α5β1 dyad. Indeed, platelets, the membrane fragments of bone-marrow megakaryocytic cells, harbor the ligand, CD154 and most of its receptors, CD40, and integrins αIIbβ3, α5β1, and αvβ3 [48,122,123]. As mentioned above, the interaction of CD154 with CD40 [124], as well as with αIIbβ3 integrin [10,84] on the platelet surface, was shown to potentiate or, depending on the concentration, induce platelet activation, adhesion, and/or aggregation. A recent study outlined α5β1 as another receptor involved in platelet activation upon its ligation to sCD154 [125]. Indeed, the use of specific blocking antibodies against the α5β1 integrin inhibited sCD154-induced platelet activation. The authors suggested an interplay between the various CD154 receptors on platelets and approved of a previous theory made by our group on the capacity and possibility of simultaneous binding of CD154 to multiple receptors expressed on the surface of the same cell. This point is based on data demonstrating that CD154 interacts with its various receptors via distinct residues [86] and the fact that CD154 exists and is biologically active as a trimeric molecule [17,18]. Such argument is of high importance in most CD154-mediated responses, given the diversity of expression patterns of CD154 receptors. A synergistic, additive, or even an inhibitory interaction could result from such a simultaneous interaction.

#### 4.4.2. Role of the CD154–α5β1 Interaction in Cell Survival

**Figure 3:**
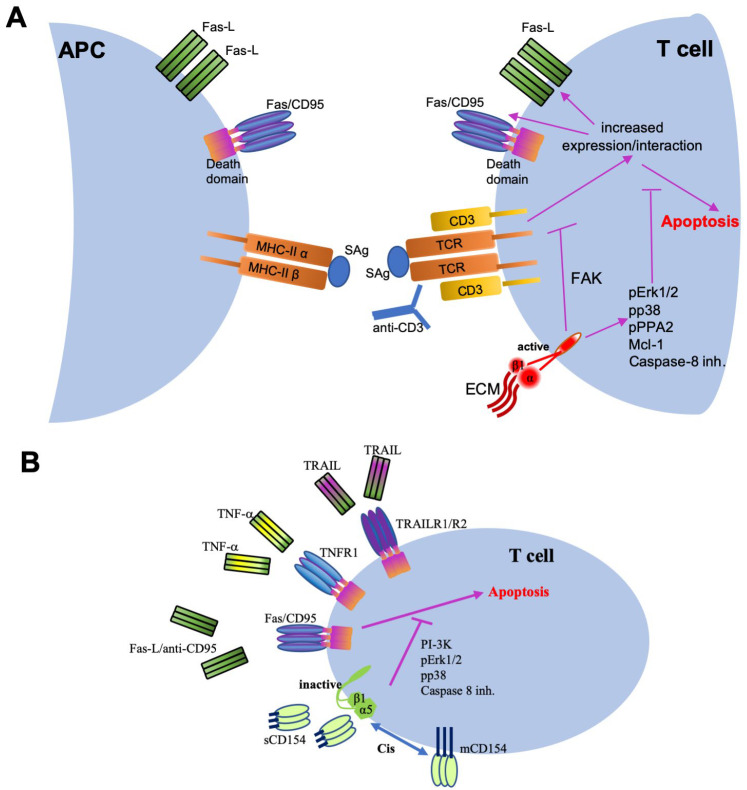
Mechanistic of the apoptosis-inhibiting role of β1 integrin–ECM and α5β1–CD154 dyads: (**A**) activating TCR/CD3 via binding to Sag–MHC–II complex (which binds to the variable region of the TCR β chain and activates T cells without being processed by APCs) or to anti-CD3 Abs, in a process termed activation-induced cell death (AICD), enhances surface expression of Fas and Fas ligand on T cells, thus promoting cell apoptosis. Binding of extracellular matrix (ECM) ligands or activating antibodies to β1 integrins abrogates this AICD by inducing signaling events such as activation of FAK, ERK1/2, and PPA2 and maintaining the survival signal, Mcl-1, leading to caspase-8 inhibition; (**B**) binding of sCD154 to α5β1 integrin inhibits apoptosis induced by Fas–FasL interaction, as well as by other death signals—namely, TNF-α and TRAIL, interacting with their respective receptors via activating survival signals including PI-3K, ERK1/2, and p38 to also lead the inhibition of caspase-8 cleavage. The α5β1 is capable of interacting with sCD154, as well as with mCD154, but in a cis-manner of binding (when both ligand and receptor are expressed on the surface of the same cell). Abbreviations: APC, antigen-presenting cell; ERK, extracellular-signal-regulated kinase; FAK, focal adhesion kinase; Mcl-1, myeloid cell leukemia-1; MHC-II, major histocompatibility complex II; PI-3K, phosphoinositide-3 kinase; PPA2, protein phosphatase A2; SAg, superantigen; TNF-α, tumor necrosis factor-α; TNFR1, TNF receptor 1; TRAIL, TNF-related apoptosis-inducing ligand; TRAILR1/R2, TRAIL receptors R1 and R2; TCR, T-cell receptor.

*The implication of β1 integrins in abrogating apoptosis:* Interesting findings have demonstrated an important role for β1 integrins in promoting the survival of immune cells, particularly T cells, of normal or malignant nature in chronic inflammatory diseases, as well as hematologic malignancies (Figure 3) [126,127,128,129,130,131,132]. Stimulating β1 integrins of T cells was shown to enhance their proliferative processes including activation of their focal adhesion kinase (FAK) and its downstream effectors including PI-3K and Akt [133]. FAK in T cells has also been described as a contributor to an important immunoregulatory process, termed activation-induced cell death (AICD) (Figure 3A) [134]. Upon antigen stimulation of T cell receptors (TCRs) of primed T cells or T-cell activation with anti-CD3 or PMA/ionomycin, T cells upregulate their gene expression of Fas/Fas ligand and are, thus, more prone to Fas-induced apoptosis [135,136,137]. The β1 integrins and their FAK have been reported as important regulators of this process. Indeed, the α2β1 integrin by interacting with its collagen ligand or with activating monoclonal antibodies was shown to abrogate AICD in T cell lines via activation of their FAK and downregulation of Fas ligand mRNA [138]. Another member of β1 integrins, α5β1, was also shown to be part of this T-cell protection squad. Indeed, α5β1 was reported to enhance the AICD-inhibiting as well as the proliferation-stimulating effects of TGFβ on CD8-positive T cells [139].

In malignant T cells, the interaction of β1 integrins with ECM components is a critical step, both for their survival as well as their spread and migration. Acute lymphoblastic leukemia (ALL) T cells exhibited an increased survival rate and an enhanced migration upon stimulating their α2β1 integrins with collagen type I in a mechanism dependent upon ERK1/2 MAPK activation and maintenance of the Bcl-2 family member, prosurvival protein, Mcl-1 [140]. Malignant T cells were also shown to develop resistance to doxorubicin-induced chemotoxicity upon ligation of their α4β1 and α5β1 integrins. Engaging the α4β1 integrin enhanced Ca^2+^ influx via an association between a juxtamembrane cytoplasmic region of its α4 chain and the Ca^2+^ binding protein, calreticulin [141]. In the same line of work, the collagen–α2β1 interaction was reported to implicate additional signaling events in its antiapoptotic function in T cells—namely, phosphorylation of protein phosphatase 2A and inhibition of caspase-8 activation [142].

Direct evidence of the implication of β1 integrins in promoting the persistence of T cells in chronic inflammatory diseases was reported in a recent study outlining the abrogation of their Fas-induced apoptosis upon exposure to the synovial fluid of RA patients in a p38 MAPK/caspase-8 complex-dependent mechanism [126]. Treating this system with anti-β1 integrin antibodies reversed the response back to apoptosis. On the other hand, transfecting T cells with an auto-active form of β1 enhanced p38 phosphorylation, further confirming the role of β1 integrin in the p38-mediated persistence of T cells [126]. These studies outlined the role of β1 integrins in cell survival involving apoptosis-inhibiting mechanisms rather than via enhancing mere adhesion to ECM.

*The CD154–α5β1 dyad in T cell survival:* With the identification of sCD154 as a new ligand for α5β1, an interesting line of research further promoted the antiapoptotic role of the CD154–α5β1 dyad (Figure 3B). Preventing apoptosis of cells and allowing their sustained presence and function is one of the main actors in the pathogenesis of inflammatory diseases where persistent inflammatory cells would aggravate the condition and of malignant disorders where stubborn cancerous cells would promote cancer perseverance and even invasion. Indeed, our recent results showed that sCD154 is capable of binding to several ALL T cell lines via their α5β1 integrin inducing the activation of their p38, ERK, and PI-3K/Akt [143]. We also demonstrated that treatment of these malignant cells with sCD154 entirely abrogated their Fas-induced cell death, in a mechanism involving the above signaling pathways mainly PI-3K activation, and also a decreased cleavage of caspase-8 (Figure 3B) [143]. These findings outline an interesting and novel role of CD154 as a player in the pathogenesis of cancer and, more specifically, hematological ones involving T cells.

The antiapoptotic role of CD154 was further explored with other death signals. Our group demonstrated that this prohibitory effect of CD154 was not restricted to Fas-induced cell death but was also directed against apoptotic events induced by other death signals [32]—namely, TNF-α and TNF-related apoptosis-inducing ligand (TRAIL) (Figure 3B) [144,145]. Given these observations, we then demonstrated that the antiapoptotic effect of CD154 is also seen in human primary T cells and, more importantly, exhibited in CD4-positive than CD8-positive T cells, suggesting that α5β1 integrin engagement with CD154 could be a critical pathway contributing to the survival and persistence of effector T cells in inflammatory diseases [32].

These exciting observations opened the door for new interpretations of already existing data as to the implication of CD154 and β1 integrins in the pathogenesis of inflammatory conditions. An interesting example here is a study by Nakayamada et al., which might be highly suggestive of a role for the CD154–α5β1 integrins in SLE development. Peripheral T cells from SLE patients with active disease were shown to overexpress β1 integrins on their surface, compared with T cells from normal subjects. Activating β1 in these cells promoted their proliferation and activation, the latter assessed by an upregulated CD154 cell surface expression [129]. Interestingly in this matter, even though not discussed in the study itself, the overexpressed CD154 in these SLE T cells could be playing a role in their persistence by acting on α5β1 integrins also expressed herein.

All these observations were demonstrated upon the interaction of α5β1 with soluble CD154. As a matter of fact, and as mentioned above, most lines of evidence of bindings between integrins and CD154 were revealed using the soluble form of the latter [10,11,12,13,14]. The next evident step was to evaluate the binding of membrane-bound CD154 to α5β1, more particularly to demonstrate their effect on apoptosis in T cells, cells where both the ligand and the receptor are expressed on the surface. The interaction of membrane-bound CD154 with α5β1 on the surface of T cells was shown to be solely undertaken in a cis-fashion (Figure 3B), i.e., when both the ligand and the receptor are expressed on the same cell as opposed to a trans-interaction when the ligand and the receptor are expressed on the surface of two different cells. The cis-binding on the cell surface was revealed for other ligand–receptor couples and protein–protein interactions [146,147,148,149]. Its role might be to secure the sequestration of the ligand by the receptor, thus decreasing its availability to interact with other receptors expressed on other cells as a form of an immune checkpoint. By binding CD154, α5β1 confiscates the ligand to its antiapoptotic function and leaves little CD154 available to bind to CD40, for instance, expressed on the surface of other cells [32].

Altogether, these data demonstrate the significant role of the α5β1 integrin as a receptor for CD154 in immune responses, inflammation, cancer, and even hemostasis. Defining all these novel functions of α5β1 puts it on the list of CD154-related targets for the development of treatment/prevention approaches.

### 4.5. The αVβ3 Integrin as a Receptor for CD154

The αvβ3 integrin, another member of the RGD-binding subfamily of integrins, with a vast range of ligands from ECM proteins and adhesion molecules to growth mediators [150], is involved in attachment to the cytoskeleton, as well as in the survival, proliferation, and motility of various types of cells including angiogenic ECs and malignant cells [151,152]. This integrin has recently been identified by the group of Takada as a new receptor for CD154. Researchers reported that CD154 is capable of binding to soluble αvβ3 in the presence of Mn^2+^ cations and, therefore, the active form of the molecule but in a KGD-independent manner [13]. It is important to mention here that while describing α5β1 as a receptor for CD154, our group reported the inability of the ligand to bind to αvβ3. The reason could be attributed to the inactive state of this latter, as it was in the presence of no cations in the solution [12,13]. Further elaboration of the CD154 binding region to αvβ3, using a simulation docking model, revealed such region to be within the monomeric CD154 at the interface of its trimeric structure, overlapping that involved in α5β1 binding and important for proper interaction of CD154 with its CD40 receptor.

Some CD154 missense mutations, usually reported under HIGM conditions, were shown to abrogate the binding of CD154 to both α5β1 and αvβ3 integrins [13]. The same authors later described on αvβ3 as well as on α5β1 and α4β1 integrins, an allosteric site termed site 2, in distinction from the RGD-binding site, site 1, as involved in the binding of these integrins to CD154. The interaction of CD154 with αvβ3, and with α5β1 and α4β1 in this matter, induces integrin activation and subsequent signal transduction and response [14].

Awaiting a thorough investigation of the biological significance of a CD154–αvβ3 interaction and its underlying signaling events, this dyad is probably at play in vascular or cancer settings given the high expression of both the ligand and receptor at this level [13]. It is worth noting at this point that αvβ3 integrin, in spite of being a receptor for CD154 and present on the surface of platelets just like CD40 and other of its integrin equals, is not involved in sCD154-induced activation of platelets [125].

### 4.6. The α4β1 Integrin as a Receptor for CD154

The most recently identified CD154 receptor is the α4β1 integrin [14]. This integrin, also known as very late antigen-4 (VLA-4), is widely expressed in immune cells including lymphocytes, natural killer cells, monocytes, macrophages, eosinophils, basophils, etc. [153]. It is implicated in homing of these cells, their migration, differentiation, and activation. Natural ligands of α4β1 include VCAM-1, mucosal addressing cell adhesion molecule (MAdCAM) as well as fibronectin [154]. Recent data have demonstrated the capacity of CD154 to “allosterically” bind to α4β1, as well as to α5β1 and αvβ3. Indeed, in addition to the usual RGD-binding sites on these integrins, what is termed site 1, a second region (site 2) with allosteric properties was shown to be also implicated in the interaction of integrins with inflammatory ligands such as Fractalkine, the stromal cell-derived factor 1, and phospholipase A2 type IIA [155,156,157]. CD154 was shown to bind to α4β1, α5β1, and αvβ3 via their site 2 as well, as mentioned above. The interaction of the ligand with site 2 is implicated in some HIGM conditions and is capable of influencing CD154–CD40 interaction [14].

A deeper investigation of the biological consequence of the CD154–α4β1 interaction is called for. We expect the CD154–α4β1 to exert highly significant functions, given the importance of both ligand and receptor in numerous aspects of cellular response (adhesion, migration, differentiation, activation, survival, etc.).

## 5. Conclusive Remarks

Integrins, major players in numerous cellular processes and involved in various aspects of human health and pathological states, are now identified as receptors for the co-stimulatory, immunomodulating, and pro-inflammatory molecule, CD154. Describing this new port of entry by which integrins are contributing to human health and disease via their interaction with CD154 constitutes a braking point for the identification of novel therapeutic targets and the development of therapeutic approaches for the treatment of integrin/CD154-mediated diseases. Abrogating the CD154–Mac-1 interaction has been successfully evaluated as a therapeutic tool in atherosclerosis and graft rejection. While one of the functions of the α5β1–CD154 dyad is promoting tumor cell survival in the context of cancer and enhancing inflammation as well as T-cell persistence in autoimmune conditions, such an axis represents a promising therapeutic target in these diseases, with minimal collateral side effects. Indeed, therapeutic strategies directed against the interaction of integrins specifically with their ligand CD154 could provide a better treatment approach for a plethora of inflammatory, autoimmune, and malignant diseases.

## Data Availability

Not Applicable.
